# Oxidized Carbon Black: Preparation, Characterization and Application in Antibody Delivery across Cell Membrane

**DOI:** 10.1038/s41598-018-20650-4

**Published:** 2018-02-06

**Authors:** Kittima Amornwachirabodee, Nattapol Tantimekin, Porntip Pan-In, Tanapat Palaga, Prompong Pienpinijtham, Chonlatip Pipattanaboon, Thanyada Sukmanee, Patcharee Ritprajak, Promchat Charoenpat, Pannamthip Pitaksajjakul, Pongrama Ramasoota, Supason Wanichwecharungruang

**Affiliations:** 1 Department of Chemistry, Faculty of Science, Chulalongkorn University, Bangkok, 10330 Thailand; 2 Center of Excellence on Petrochemical and Materials Technology, Chulalongkorn University, Bangkok, 10330 Thailand; 3 Nanotec-Chulalongkorn University Center of Excellence on Food and Agriculture, Chulalongkorn University, Bangkok, Thailand; 4 Department of Microbiology, Faculty of Science, Chulalongkorn University, Bangkok, 10330 Thailand; 5 Center of Excellence for Antibody Research, Faculty of Tropical Medicine, Mahidol University, Bangkok, 10400 Thailand; 6 Department of Microbiology, and RU in Oral Microbiology, Faculty of Dentistry, Chulalongkorn University, Bangkok, 10330 Thailand; 7 Center of Excellence for Antibody Research, and Department of Social and Environmental Medicine, Faculty of Tropical Medicine, Mahidol University, Bangkok, 10400 Thailand; 8 Center of Excellence in Materials and Bio-Interfaces, Chulalongkorn University, Bangkok, 10330 Thailand

## Abstract

Modulating biomolecular networks in cells with peptides and proteins has become a promising therapeutic strategy and effective biological tools. A simple and effective reagent that can bring functional proteins into cells can increase efficacy and allow more investigations. Here we show that the relatively non-toxic and non-immunogenic oxidized carbon black particles (OCBs) prepared from commercially available carbon black can deliver a 300 kDa protein directly into cells, without an involvement of a cellular endocytosis. Experiments with cell-sized liposomes indicate that OCBs directly interact with phospholipids and induce membrane leakages. Delivery of human monoclonal antibodies (HuMAbs, 150 kDa) with specific affinity towards dengue viruses (DENV) into DENV-infected Vero cells by OCBs results in HuMAbs distribution all over cells’ interior and effective viral neutralization. An ability of OCBs to deliver big functional/therapeutic proteins into cells should open doors for more protein drug investigations and new levels of antibody therapies and biological studies.

## Introduction

Remarkable advances in an understanding of signaling networks of disease progression together with developments of affinitive macromolecules in the past two decades, have made the interfering of biomolecular networks one of the most exciting researches and therapeutic means^1–3^. Various specific affinitive macromolecules including RNA/DNA aptamers, siRNA, peptides and proteins have been tested with positive results^4–6^. In addition to many therapeutic applications, synthetic antibodies have been tailored as tools for various intracellular targets (intrabodies)^7^, and have been successfully used for misfolded protein recognition^8^, sensing protein conformation^9^, and *in vivo* homing^10^. Many of these applications require the transport of proteins into cells. In addition to the use of cell penetrating peptides which require chemical coupling, and conventional liposomes which are unstable, a simple reagent that can effectively bring small peptides and big proteins into cells is, therefore, being needed^11,12^. Apart from minimal toxicity, ideal reagents should possess simplicity during usages, and should be effective in delivering cargoes into cells without being destroyed by the commonly encountered endosome/lysosome pathway^13,14^.

Our involvement in this area started from our preparation of the oxidized carbon nanospheres (OCNs) that possess excellent ability to bring macromolecules into cells^15–17^. Although the previously reported OCN can be effectively used as a delivery reagent to bring matters into cells, there are many limitations on the OCN preparation. An average synthesis yield of OCNs from graphite or graphene is limited to 8%. Its synthesis is non-trivial regarding the generation of side-reaction products such as oxidized carbon nanotubes and graphene oxide sheets, thus extensive multi-step centrifugal purification process is needed. In order to minimize these drawbacks, we have been working on a better method to prepare the OCNs. Finally, instead of getting the exact OCNs by a different method, we have obtained the oxidized carbon black particles (OCBs). This new OCB material which can be easily derived from commercially available carbon black, is able to effectively deliver cargoes through the cell membrane. More importantly, the transport of macromolecules into cells by the OCBs can be achieved without an involvement of a cellular endocytic process. This paper shows the synthesis and characterization of OCBs. Their ability to induce leakages on phospholipid bilayer membranes of artificial cells (cell-sized liposomes) and real cells is demonstrated. We also show here an application of OCBs on the sending of therapeutic antibodies into cells to perform intracellular viral neutralization.

## Results

### Synthesis and characterization of OCBs

**Figure 1:**
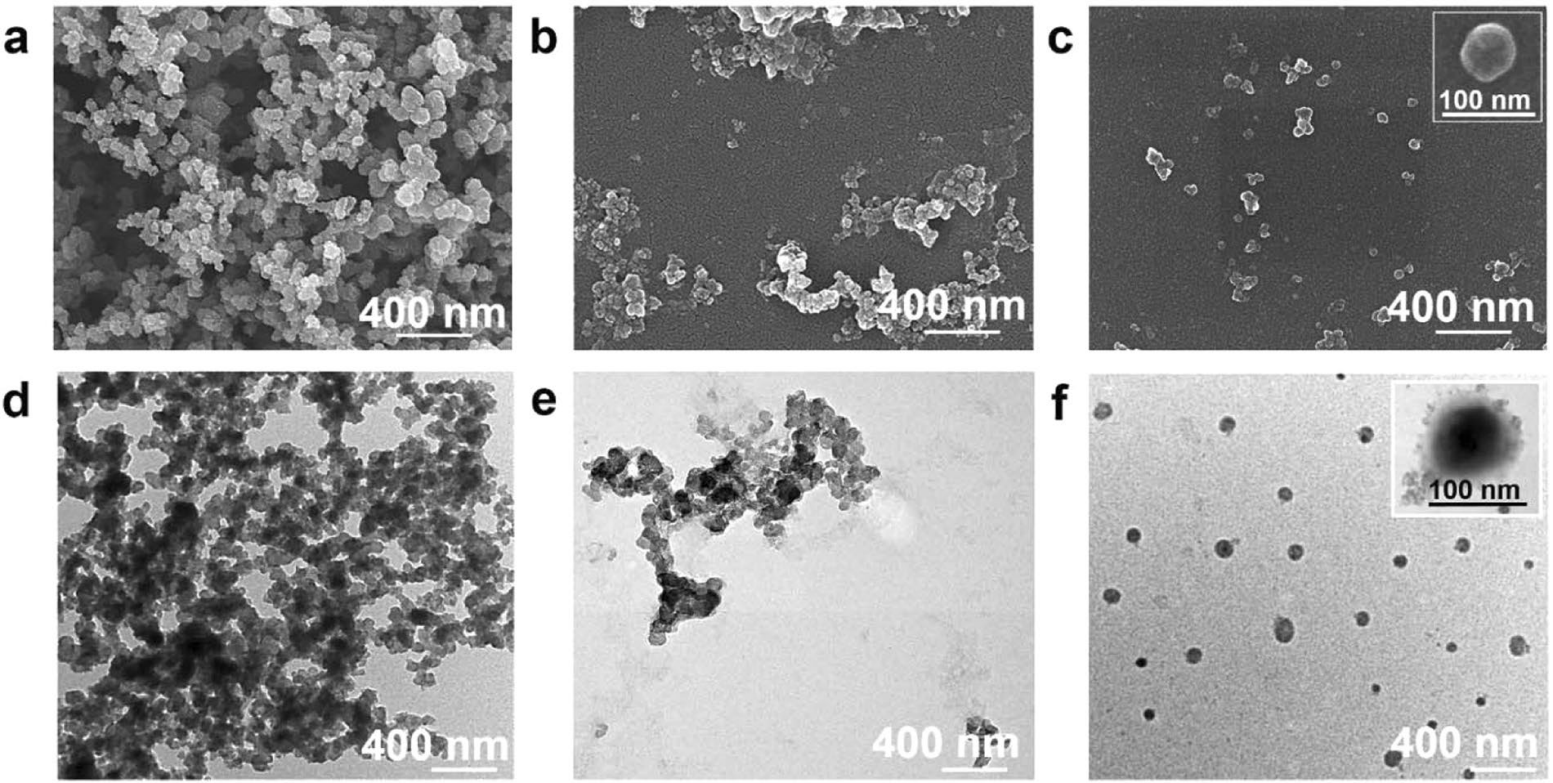
Morphology characterization of starting carbon black (CBs) and oxidized carbon black (OCBs). SEM (**a**,**b** and **c**) and TEM (**d**,**e** and **f**) images of the CBs (**a** and **d**) and the OCBs obtained from reactions at the CBs to KMnO_4_ weight ratios of 0.5:6 (**b** and **e**) and 0.3:6 (**c** and **f**).

The starting carbon black particles (CBs) do not disperse in water and their scanning electron microscopic (SEM) and transmission electron microscopic (TEM) images show that they are aggregates of many spherical particles. (Fig. 1). Reacting the CBs with NaNO_3_, H_2_SO_4_ and KMnO_4_, resulted in a black suspension of the water dispersible oxidized carbon black nanoparticles (OCBs). The suspension showed no precipitation even after sitting for 1 year (Supplementary Information, Figure S1). Among the three weight ratios of CBs to KMnO_4_ (0.5:6, 0.3:6 and 0.1:6) experimented during the optimization of the preparation process, the reaction at 0.3:6 ratio gave the highest yield (18%) of water dispersible OCBs. SEM and TEM images reveal that the OCBs obtained from the oxidation at the 0.3:6 ratio possess less aggregation among particles than those obtained at the 0.5:6 ratio (Fig. 1, see also Table S1 in Supplementary Information). Hydrodynamic size (obtained from dynamic light scattering, Supplementary Information, Table S1) of OCBs obtained from the 0.3:6 ratio (127 ± 0.51 nm, PDI 0.18) is smaller with narrower size distribution than that obtained from the 0.5:6 ratio (255 ± 2.17 nm, PDI 0.33). The prepared OCBs possess the zeta potentials of −33 to −34 mV. In contrast, reaction at CB: KMnO_4_ of 0.1:6 gave a clear colorless solution with no particulate product.

The OCBs obtained from reaction with CB: KMnO_4_ of 0.3:6 were subjected to structural analysis. X-ray photoelectronic spectra (XPS) show an increase in oxygen content upon the oxidation of CBs into OCBs (Figure S2a1 and b1 in Supplementary Information); C1s and O1s spectra of CBs show minute amounts of C-O and C=O (Supplementary Information, Figure S2a1,2,3), C1s spectrum of OCBs shows high intensity peaks at the binding energy (BE) of 283.9, 285.3, 286.2, 287.5, and 289.7 eV which correlate well to the C−C, C=C, C−O (from C−O−C and C−OH), C=O, and COOH functional groups, respectively (Supplementary Information, Figure S2b2); the O1s spectrum of the OCBs also shows high intensity peaks at the binding energy of 532.8, 531.6, and 530.9 eV which correspond to the C−O (from C−O−C and C−OH), C=O and COOH functional groups, respectively (Supplementary Information, Figure S2b3). The CHO elemental analysis (EA) detected only carbon in the starting CBs, whereas C, H and O at the molar ratio of 1.0: 0.27: 0.64 could be detected in the OCBs. In short, elemental analyses show an increase in oxygen content upon the oxidation of CBs into OCBs, and the –COOH, -OH, C=C, C-O-C, C=O functionalities at the particle surface can be deduced from the XPS spectra of the OCBs.

UV absorption spectrum of the OCBs (Supplementary Information, Figure S3a) shows maximum absorption at 244 nm (n → π* of C=O) with broad extension up to 600 nm (the π → π* transition of C=C conjugated carbon networks). FTIR spectrum of OCBs (Supplementary Information, Figure S3b) shows a broad O-H stretching at 3338 cm^−1^, C=O stretching and C=C stretching at 1600–1800 cm^−1^, C-O stretching/C-H bending/O-H bending at 1100 cm^−1^–1410 cm^−1^. Raman spectrum of the starting CBs shows a typical G band (also called graphite peak) at ~1590 cm^−1^, D band (also known as sp^2^ carbon disorder-induced peak) at ~1360 cm^−1^, with almost undetectable 2D or G′ band (disordered sp^2^ planes) at 2500–3400 cm^−1^. A slightly blue shift of the D-band in the OCBs spectrum, as compared to that of the CBs, can be observed (Supplementary Information, Figure S3c). In addition, the D band to G band peak area ratio increases from 1.25 for the starting CBs to 1.56 for the OCBs. Multiple broad 2D bands are also obvious in the Raman spectrum of OCBs, but are undetectable in the CB spectrum.

Lastly, stability of the particles’ surface was analyzed through the monitoring of hydrodynamic size of the OCBs, and the result showed no size change during the 5 h monitoring period (Supplementary Information, Figure S3d), agreeing well to their never-settling aqueous colloidal nature described earlier.

### Penetration of OCBs into cell-sized liposomes

#### Penetration of OCBs into liposomes

**Figure 2:**
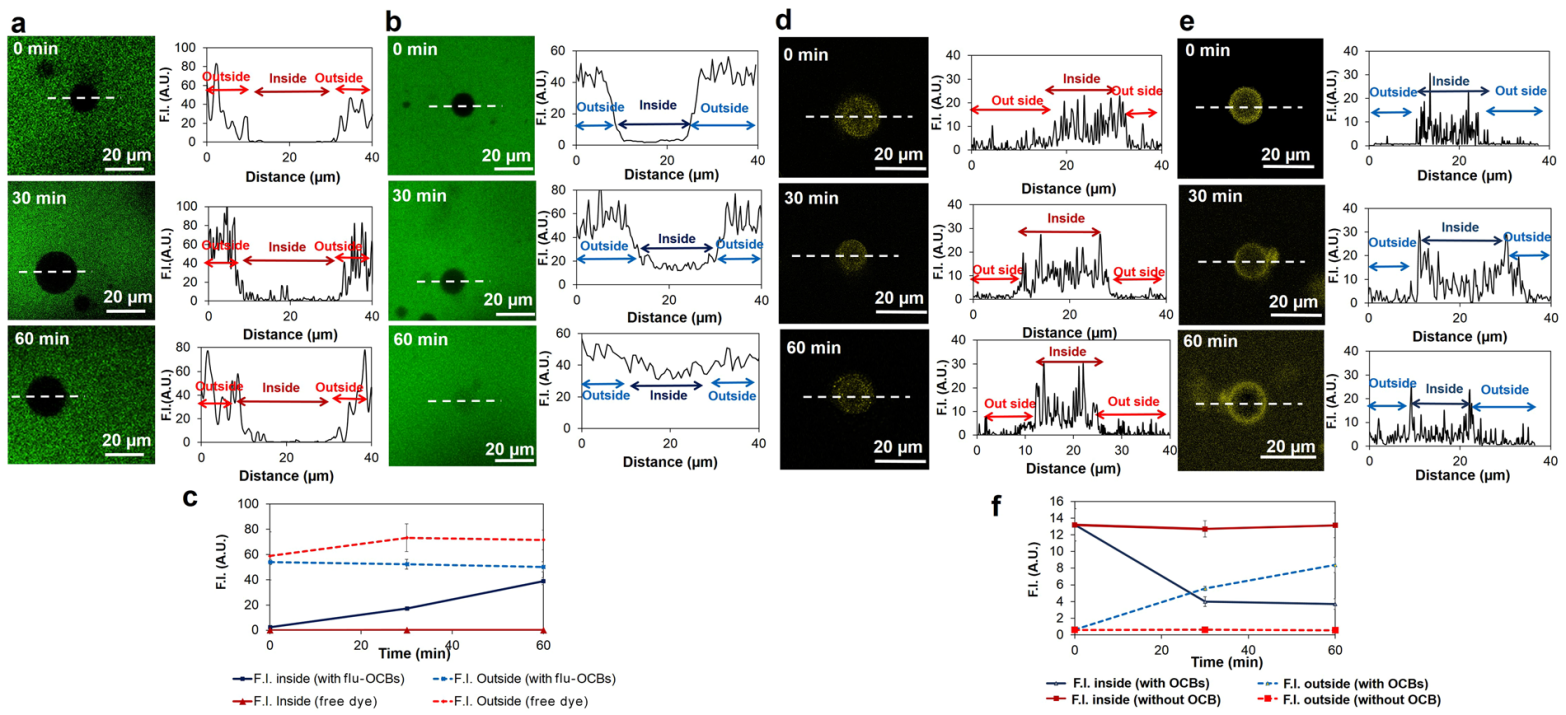
Liposome leakage. *Penetration of flu-OCBs into liposomes* (**a**,**b** and **c**): (**a**) Fluorescence images of liposomes after being incubated with flu (free dye molecules, in green) for 0, 30 and 60 min (left) accompanied with plots of fluorescence intensity (F.I.) along the dotted line of the corresponding liposomes (right), (**b**) Fluorescence images of liposomes after being incubated with flu-OCBs (in green) for 0, 30 and 60 min (left) accompanied with plots of F.I. along the dotted line of the corresponding liposomes (right), (**c**) Plots of F.I. at the inside and outside of the liposomes, as a function of incubation time (shown as mean ± SD). *Anthocyanin leak from liposomes* (**d**,**e** and **f**): (**d**) Fluorescence images of anthocyanin (pseudo-color, yellow) filled liposomes after being incubated with water for 0, 30 and 60 min (left) accompanied with plots of F.I. along the dotted line of the corresponding liposomes (right), (**e**) Fluorescence images of anthocyanin (pseudo-color, yellow) filled liposomes after being incubated with OCBs for 0, 30 and 60 min (left) accompanied with plots of F.I. along the dotted line of the corresponding liposomes (right), (**f**) Plots of anthocyanin F.I. at the inside and outside of the liposomes after water addition (without OCB) or OCBs addition (with OCBs), as a function of incubation time (shown as mean ± SD).

The fluorescein-labeled OCBs (flu-OCBs) were incubated with cell-sized liposomes and confocal laser fluorescence microscope (CLFM) was used to observe changes. Firstly, liposomes were incubated with fluorescein (flu) as a control, no fluorescence signal was observed at the inside of the liposomes (Fig. 2a, 0–60 min). Secondly, liposomes were incubated with flu-OCBs (Fig. 2b). At the beginning, the fluorescence signal inside of liposome was undetectable (Fig. 2b, 0 min). However, after 30 min, the fluorescence signal at the inside of the liposome was obvious, and its intensity kept increasing along the incubation time (Fig. 2b and c).

#### Liposome leak induced by OCBs

Liposomes filled with anthocyanin were prepared, then OCBs were introduced to the outside of the liposomes, and the fluorescence signals of anthocyanin at the inside and outside of liposomes were monitored. At first, the fluorescence of anthocyanin at the outside of the liposomes was undetectable (Fig. 2e, 0 min). Further incubation resulted in a decrease of anthocyanin signal at the liposomes’ interior and an increase of that at the outside of the liposomes (Fig. 2e and f). At 30 min, fluorescence at the outside of the liposomes was very obvious. In the control experiment (no OCB), fluorescence signal was never observed at the outside of the liposomes (Fig. 2d and f). At the concentration of the OCB used in this experiment (100 mg/L), the numbers of liposomes left in the systems after 60 min incubation were the same for both the system with added OCBs and the system without OCB addition.

#### Adsorption of materials onto OCBs

Adsorptions of various molecules onto OCBs were monitored through the change of OCB size. Among the cholesterol, bovine serum albumin (BSA) protein, dioleoyl L-α-phosphatidylcholine (DOPC), egg phospholipids and antibody (HuMAbs), the egg phospholipids gave the highest size change to the particles whereas antibody showed no significant effect on OCB’s size (Supplementary Information, Figure S4).

### *In vitro* cytotoxicity and immunogenicity of OCBs

The cytotoxicity of OCBs was investigated by an MTT method on macrophage-like cell line (RAW 264.7, purchased from ATCC), human epidermoid cervical carcinoma cell line (CaSki, purchased from ATCC) and African green monkey kidney cell line (Vero, purchased from ATCC) at cell density of 1 × 10^4^ cells in culture medium. Cells were cultured in the presence of OCBs at concentrations of 0–100 mg/L for 48 h, and cell viabilities of more than 80% were observed in the presence of ≤3.2, 10 and 25 mg/L of OCBs for RAW 264.7, CaSki and Vero cells, respectively (Supplementary Information, Figure S5a). We further monitored the viability of RAW 264.7 cells after being incubated with various concentrations of OCBs for 1 week (here the starting cell density was 1 × 10^3^ cells), and the cell viability of more than 80% was observed at concentration of OCBs of ≤1.0 mg/L (Supplementary Information, Figure S5b).

The cytotoxicity of OCBs was also tested in a primary dendritic cell, a murine bone marrow-derived dendritic cell (BM-DC), using an apoptosis detection assay. The living cells were negative for Annexin V and 7AAD staining, while the apoptotic cells were positive for both staining. OCBs demonstrated the non-toxicity in BM-DCs as the percentages of the living and apoptotic cells exposed to OCBs (at 0–100 mg/L) was approximately 80% and 20%, respectively (Supplementary Information, Figure S5c).

We also investigated the immunogenicity of OCBs with the BM-DCs by performing flow cytometric analysis of the expression of CD80, CD86 and MHC class II activation markers (Supplementary Information, Figure S6a and b). OCBs, at all concentrations (1–100 mg/L), did not activate BM-DCs as there was no increased expression of all the monitored activation markers.

### Cellular uptake of OCBs and protein delivery

#### Monitoring of cellular uptake in living cells

**Figure 3:**
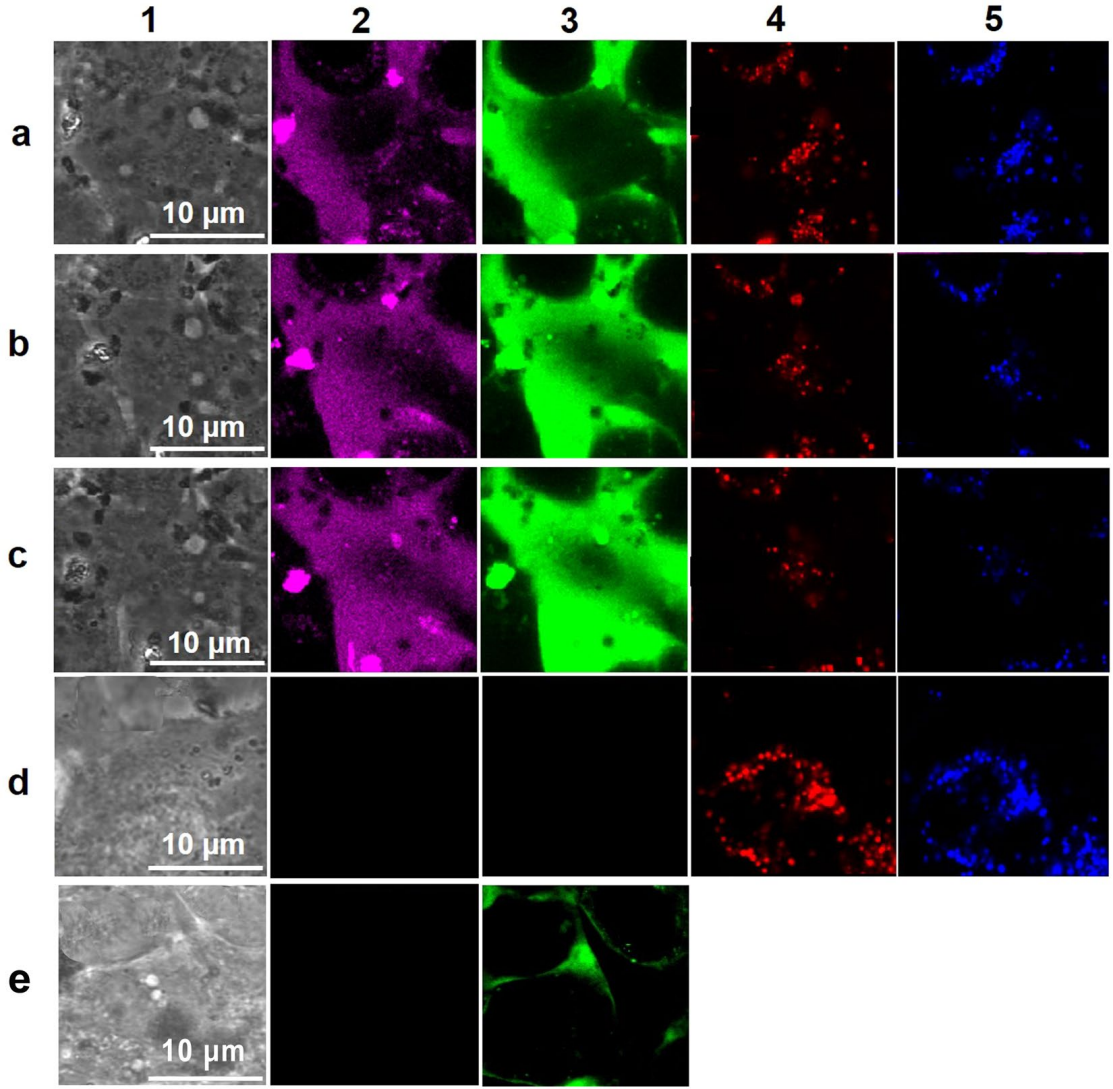
Uptake of flu-lysozyme protein and cou-OCBs into CaSki cells. CLFM images of living CaSki cells after being incubated with flu-lysozyme protein plus cou-OCBs for 15 (row **a**), 30 (row **b**) and 45 (row **c**) min. Control cells (no flu-lysozyme protein, no cou-OCB) are shown in row (**d**). CaSki cells after being incubated with only flu-lysozyme protein (no cou-OCB) are shown in row (**e**). Cell images under phase contrast mode are shown in column 1. Signals from cou-OCBs in magenta (pseudo-color, column 2), flu-lysozyme protein in green (column 3), early endosome in red (column 4) and lysosome in blue (pseudo-color, column 5).

We investigated abilities of the OCBs to deliver hen egg white lysozyme protein into living CaSki cells (Fig. 3). First fluorescein-labeled lysozyme protein (flu-lysozyme) and coumarin-labeled OCBs (cou-OCBs) were prepared through coupling reactions using 1-ethyl-3-(3-dimethylaminopropyl) carbodiimide (EDCI) as coupling agent. Cell images under phase contrast mode (column 1) and fluorescence signals of the CaSki cells (detected by CLFM) after being incubated with the cou-OCBs (magenta color (pseudo color), column 2) and flu-lysozyme (green color, column 3) for 15, 30 and 45 min, are shown in row a, b and c, respectively. It should be noted here that the fluorescence dyes specific to early endosomes (red color, column 4) and lysosomes (blue (pseudo color), column 5) were also used in the experiment. Fluorescence image of control cells (no flu-lysozyme protein, no cou-OCB) are shown in row d, and that of CaSki cells after being incubated with only flu-lysozyme protein (no cou-OCB, and no fluorescence dyes specific to early endosomes and lysosomes) are shown in row e. Noted that when only flu-lysozyme protein was used, endosome and lysosome tracker dyes were not used.

#### Intracellular localization

**Figure 4:**
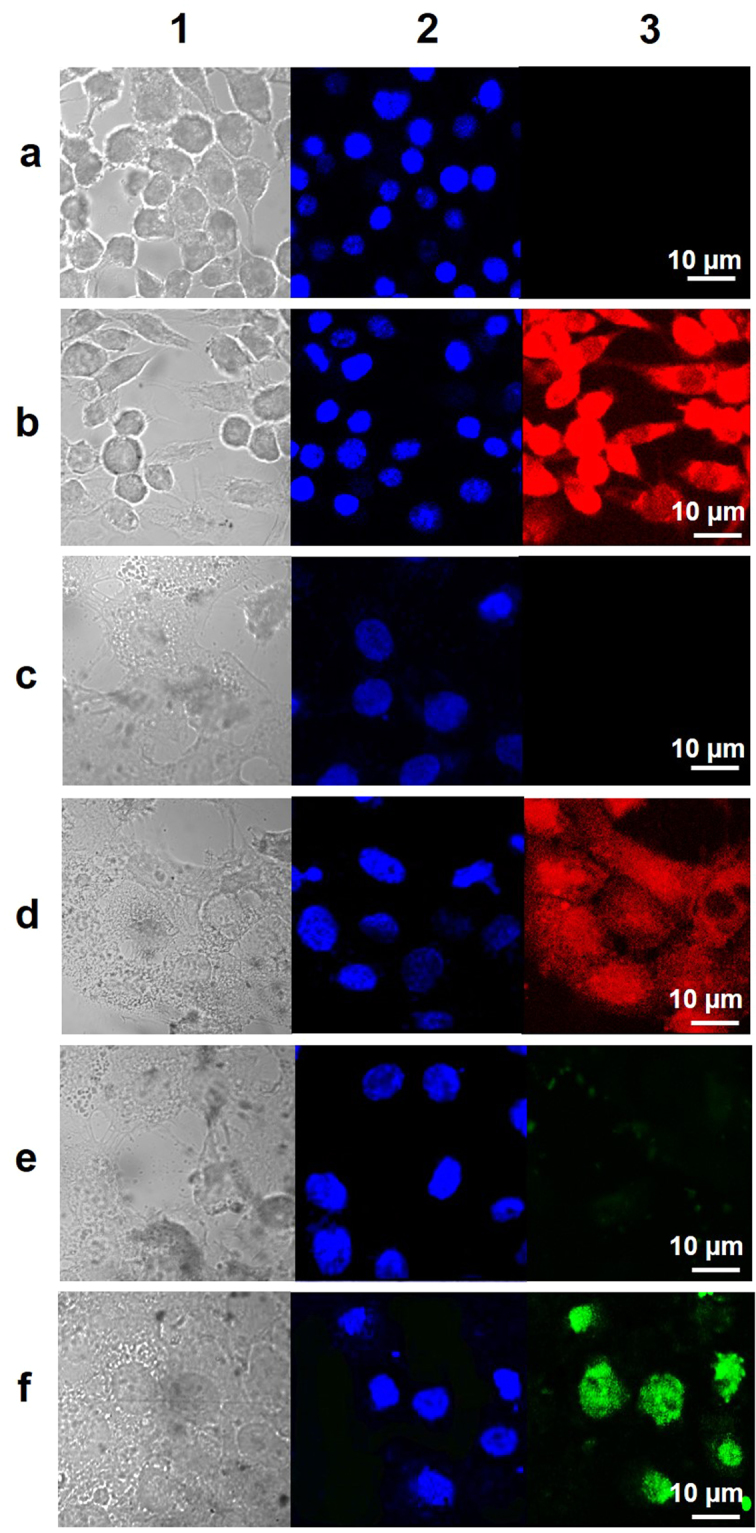
Cell membrane and nuclear membrane penetration of OCBs. CLFM images of RAW 264.7 (row **a** and **b**) and CaSki (row **c**, **d**, **e** and **f**) cells after being incubated for 240 min with media (control cells, row **a** and **c**), TAMRA-OCBs (row **b** and **d**), flu-lysozyme (row **e**), and flu-lysozyme plus OCBs (row **f**).

OCBs were first labeled with tetramethylrhodamine (TAMRA) to obtain TAMRA-OCBs. Cells (RAW 264.7 and CaSki cell lines) were then incubated with the TAMRA-OCBs for 4 h and then thoroughly washed before being subjected to the fixation process to stain cells’ nuclei with DAPI. Then the fixed cells were subjected to CLFM analysis. The fluorescence signal of the TAMRA-OCBs could be clearly detected in both cytoplasm and nucleus of the two cells (Fig. 4b for RAW 264.7 and 4d for CaSki cells). Next, we incubated the flu-lysozyme and OCBs with the CaSki cells for 4 h and subjected the cells to washing and nuclear staining processes, then the fixed cells were observed under a CLFM. The result shows no signal of the flu-lysozyme in the cells when OCB was not used (Fig. 4e). In contrast, with OCBs, obvious fluorescence signals of the flu-lysozyme could be seen in both cytoplasm and nucleus the cells (Fig. 4f).

### Delivery of dengue-HuMAbs into Vero cells

**Figure 5:**
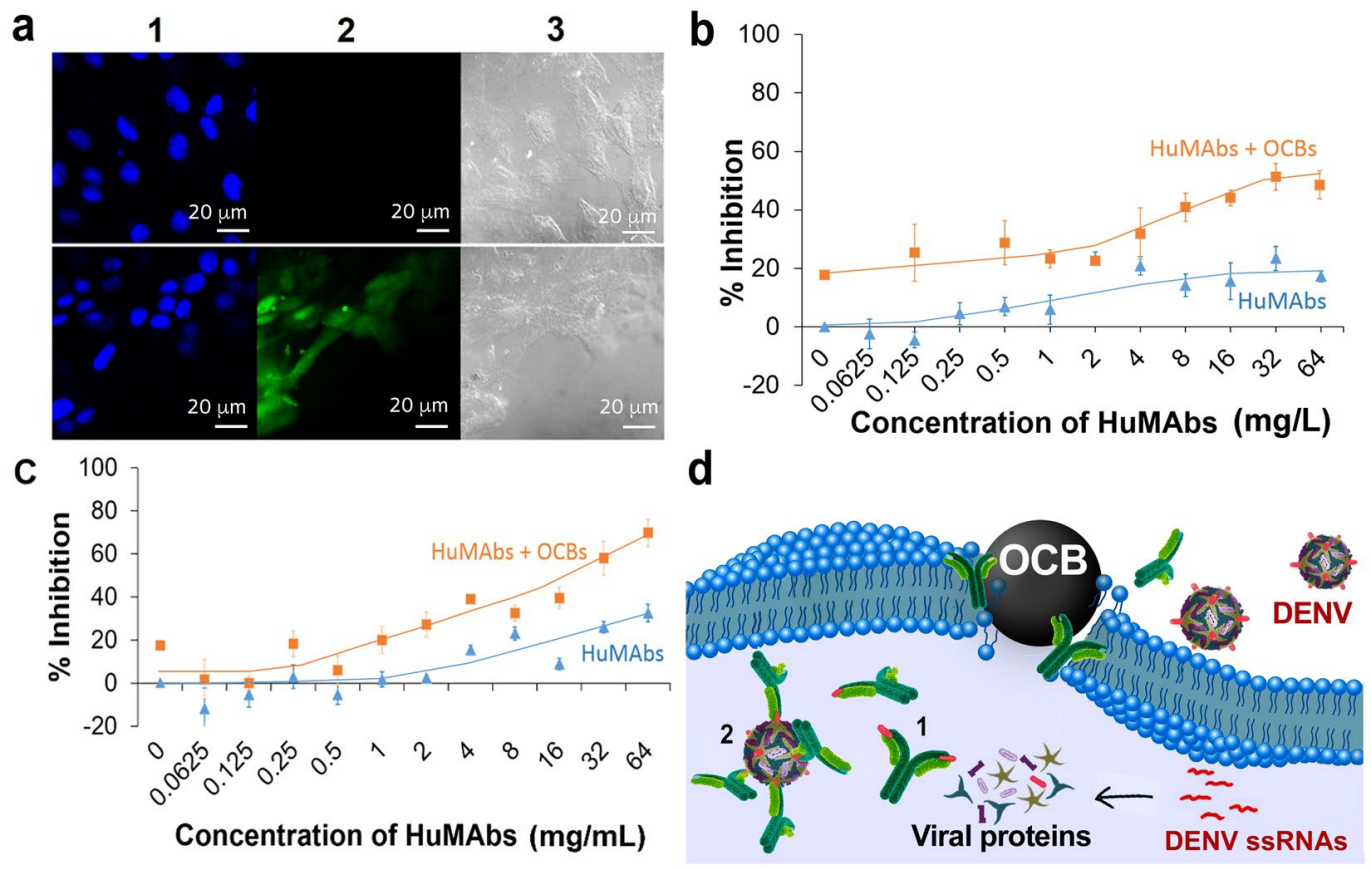
Delivery of HuMAbs into cells by OCBs and intracellular viral neutralization. (**a**) CLFM images of HuMAbs inside Vero cells: Vero cells incubated with flu-HuMAbs alone (top row), and flu-HuMAbs plus OCBs (bottom row), for 2 h; fluorescence signals from DAPI (blue, indicating nuclei) and flu-HuMAbs (green) are shown in column 1 and 2, respectively; cell images in phase contrast mode are shown in column 3. (**b**,**c**) Neutralization of viruses in Vero cells that have been infected with DENV serotype 1 (**b**) and serotype 4 (**c**) by incubating the DENV-infected cells with various concentrations of HuMAbs (alone or with 15 mg/L OCBs). (**d**) Proposed mechanisms of the intracellular neutralization of DENVs by HuMAbs; (1) HuMAbs bind to the viral envelope proteins before the proteins are assembled into viruses or (2) HuMAbs bind to the already assembled viruses before the viruses get out of the cells.

We investigated an ability of OCBs to deliver the Human monoclonal antibodies (HuMAbs) with a specific affinity towards the four DENV serotypes, into Vero cells. The HuMAbs were produced from Human hybridoma cells as described in the method section. We prepared fluorescein-labeled HuMAbs (flu-HuMAbs) using EDCI coupling reaction. The flu-HuMAbs (with and without OCBs) were then incubated with Vero cells. Without OCBs, no fluorescence signal of flu-HuMAbs could be observed inside the cells even after 4 h incubation (Fig. 5a, top row). In the presence of OCBs, flu-HuMAbs fluorescence signals could be clearly observed inside the cells after 1–2 h incubation (Fig. 5a, column 2 of bottom row).

### Viral Neutralization

Serotype 1 and serotype 4 of the DENVs were separately experimented. Vero cells were infected with the viruses and the cells were carefully and thoroughly washed to remove extracellular viruses. Then, immediately HuMAbs alone or HuMAbs mixed with OCBs were incubated with the cells. The indirect immunofluorescence assay was executed to evaluate focus-forming units (FFU). Percentages of viral inhibition were calculated from the obtained FFU^18^. Responses to the HuMAbs were different among different DENV serotypes (Fig. 5b,c). Nevertheless, neutralizations of the two serotypes were dose dependent and more pronounced in the presence of the OCBs.

## Discussion

The non-water dispersible nature of CBs implies their hydrophobic surface. SEM and TEM images indicate that the CBs are aggregates of 100 nm sized spherical particles (Fig. 1). This information agrees with the previously suggested structure of the CBs which are clusters of many spherical carbon particles linked among one another through covalent bonds or van der Waal’s forces^19,20^. By oxidizing the CBs with an appropriate amount of the oxidizing agents, we could obtain the water dispersible OCBs with acceptable yield of 18%. Too much KMnO_4_ in the oxidation process resulted in the total oxidation of CBs into CO_2_, as only clear solution with no particulate was obtained. However, too little KMnO_4_ resulted in a bigger and wider size ranged OCBs. Here the 127 ± 0.51 nm OCBs obtained from the 0.3:6 weight ratio of CB to KMnO_4_ were used for further investigation. Data from XPS, FTIR, UV and Raman spectroscopy indicate that the OCBs consist of π-conjugated networks of carbon atoms with some disordered planar carbon planes, and some epoxide, carboxyl and hydroxyl functionalities at the surface. The structural deformation of the planar carbon (sp^2^ carbon) upon the oxidation of CBs into OCBs can be confirmed with the increase of the D band as compared to the G band and the appearance of multiple broad 2D bands in the Raman spectrum of OCBs as compared to that of the CB.

It should be noted here that, comparing to the previously reported membrane penetrating agent OCNs^15–17^, which can be synthesized from graphite and graphene, the preparation of OCBs demonstrated here not only gives more than double the yield but also produces neither tube nor sheet by products, thus the multi-step centrifugation process is not needed. With such a simpler OCB preparation process, we investigated whether this newly prepared material would have an ability to bring macromolecules across lipid bilayer membrane.

To eliminate any influences from trans-membrane proteins and other active transport processes in real cells, here we investigated the penetration of OCBs across lipid bilayer membrane using cell-sized liposomes constructed from the phospholipid commonly found in membranes of living cells (DOPC)^21,22^. The result that the fluorescence intensity of flu-OCBs at the inside of the liposomes increased with the incubation time with the flu-OCBs, implies that the OCBs can penetrate across the lipid bilayer membrane into the liposomes’ interior and the extent of the penetration varies with time. Since the shape of the liposome did not significantly change after the OCB penetration, we have concluded that the penetration occurred without significant deformation of the lipid bilayer wall of the liposomes. It should be noted here that the free dye molecules (flu), could not penetrate the liposomes.

One hypothesis on the penetration of the OCBs into liposomes is that OCBs probably induce local disruption of the phospholipid bilayer and create a transient leak of the membrane. We tested this transient leak hypothesis by introducing OCBs to the outside of the anthocyanin-filled liposomes, and monitoring the anthocyanin leak from the liposomes. At first, the fluorescence of anthocyanin at the outside the liposomes was undetectable (Fig. 2d, 0 min), confirming that anthocyanin molecules were initially confined at the liposomes’ interior. Further incubation with OCBs resulted in a decrease of anthocyanin signal at the liposomes’ interior and an increase of the anthocyanin signal at the outside of the liposomes (Fig. 2d and e), thus indicating some leakages of anthocyanin from the liposomes. No leak could be observed in control experiment where there was no OCB (Fig. 2c). The data thus confirm our hypothesis that OCBs can induce transient leak of the phospholipid bilayer membrane. Since the numbers of liposomes left in the systems were not affected by the OCB addition, we have also concluded that the OCB, although can induce the liposome leak, does not induce the liposome break.

We hypothesized that some adsorption of phospholipids onto OCBs was responsible for the local disruption of phospholipid organization which led to the transient leak. To investigate on this hypothesis, we have monitored the adsorptions of proteins (BSA and antibody), cholesterol and two types of phospholipids onto the OCBs. The phospholipids show better adsorption to OCBs, comparing to the protein (Supplementary Information, Figure S4). Moreover, different lipids show different adsorption ability onto the OCBs, e.g., phospholipids are better adsorbed than cholesterol. This result, although cannot confirm that the transient leak is induced by the adsorption of phospholipids onto the OCBs, indicates that phospholipids possess good adsorption to OCBs. The good affinity of phospholipids towards OCBs agrees well to our observation under CLFM that when the fluorescence-labeled OCBs were introduced into the liposome suspension, the OCBs always moved rapidly to adhere to the liposomes’ wall and later on some would migrate to the liposomes’ interior. The result here partly supports our speculation that the transient leak of the membrane might be related to the temporary disruption of the lipid bilayer organization caused by some adsorption of the phospholipids on OCBs.

It has been known that an electroporation is a method used to bring macromolecules into cells by inducing transient pore on the cell membrane using high electrical voltage^23^. With the same rationality, it is possible that the transient leakage of lipid bilayer membrane induced by OCBs could also lead to the delivery of macromolecules into cells. Prior to the exploration of such ability, we investigated the cytotoxicity of OCBs on RAW 264.7, CaSki and Vero cells, using MTT method, to obtain concentrations of OCBs which each cell type can tolerate (≥80% survival). Different cell types showed different tolerances against OCBs (Supplementary Information, Figure S5). RAW 264.7 cells were the most sensitive among the three tested cell types. Nevertheless, at 48 h incubation, up to 3.2 mg/L of OCBs was well tolerated by the RAW 254.7 cells. In contrast to a short term incubation (48 h or less), incubation of RAW 264.7 and OCBs of various concentrations for 7 days showed negative effect on cell viability at the concentration of 3.2 mg/L or higher. The lower OCB concentrations (≤1 mg/L) were well tolerated by the RAW 264.7 cells even at 7 day exposure. This result suggests that OCBs may exhibit chronic negative impact on cell viability when used at a high concentration with longer exposure time in macrophages.

Furthermore, we also tested the cytotoxicity of OCBs in BM-DC. Since the cellular metabolism of dendritic cell is highly changed after the cell interacts with stimuli^24,25^, MTT assay may not be a reliable method to detect OCB toxicity in this cell type, an apoptosis detection assay thus was used. The result indicates relatively low cytotoxicity of the OCBs at the tested concentrations of 1–100 mg/L (Supplementary Information, Figure S5a,b and c).

A dendritic cell is an innate immune cell capable of antigen presentation and induction of adaptive immune responses^26^. To investigate the immunogenicity of OCBs, the BM-DCs were incubated with OCBs. Then CD80, CD86 and MHC class II activation marker expression were monitored **(**Supplementary Information, Figure S6a and b). OCBs, at all tested concentrations (1–100 mg/L), did not activate BM-DCs, indicating that the material is non-immunogenic. This relatively low *in vitro* cytotoxicity and non-immunogenicity of OCBs indicate the material’s biocompatibility when used at low concentrations.

We used the OCB to bring hen egg white lysozyme protein (MW of 300 kDa, a representative macromolecule) into CaSki cells. The CLFM observation of the cellular penetration was carried out in living cells. The fluorescence signals observed under CLFM after incubating the flu-lysozyme and cou-OCBs with CaSki cells (Fig. 3) visibly indicate that the protein (purple color, column 2) and the OCBs (green color, column 3) were taken up into the cells. Locations of endosomes and lysosomes are clearly unrelated to the locations of OCBs and lysozymes, implying that OCBs and lysozymes were not confined in these two subcellular organelles. These results imply that endocytosis was not involved with the cellular penetration of the two materials. In contrast, without OCB, the lysozyme could not penetrate the cells (column 3, row e). We, thus, have concluded that the OCBs can deliver protein into cells and the delivery mechanism is non-endocytic.

Because the above CLFM observation was done on living cells, nuclear staining could not be carried out. This makes the obtained cell images a little hard to understand. Therefore, we further performed the cellular penetration study on more cell types, but with CLFM observation on fixed cells. After 4 h incubation of TAMRA-OCBs with the cells, the fluorescence signal of the TAMRA-OCBs could clearly be detected in both cytoplasm and nucleus of RAW 264.7 and CaSki cells (Fig. 4b and d), indicating that the particles could penetrate both cell and nuclear membranes. Incubating the flu-lysozyme with the CaSki cells for 4 h, resulted in no signal of the flu-lysozyme in the cells when OCB was absent (Fig. 4e), and obvious fluorescence signals of the flu-lysozyme at the nucleus of the cells when OCBs were presence (Fig. 4f). We conclude that the OCBs can effectively deliver the 300 kDa hen egg lysozyme protein into the cytoplasm and nucleus of cells.

The important question to be addressed is whether the proteins delivered into cells by OCBs still possess active configuration and thus are able to perform biological function. Therefore, we selected a challenging protein delivery application; the delivery of an antibody with affinity towards a disease bearing virus, into virus-infected cells, to perform viral neutralization intracellularly. This experiment should allow us to know whether the conformation and binding affinity of the proteins are still preserved after being delivered into cells by OCBs. Another reason for selecting this application is that there is currently no report on intracellular antibody therapeutics regardless of the facts that antibodies can be engineered to catch desired viruses. In addition, antibodies can be made to specifically block various protein-protein interactions that small molecules cannot block^27–29^. Therefore, an ability to deliver therapeutic antibodies into cells will bring antibody drug therapy into a new level that would have a significant impact on human health. With the use of the relatively non-toxic and non-immunogenic OCBs, delivery of HuMAbs, with a specific affinity towards DENV, into Vero cells could be achieved, and the delivered antibodies distributed well all over the interior of the cells (Fig. 5a). The protein configuration and binding affinity of the delivered HuMAbs were preserved as effective viral neutralization could be achieved (Fig. 5b,c). In addition, improved viral neutralization could be obtained when HuMAbs were used with OCBs, comparing to when they were used alone. To the best of our knowledge, this is the first report on intracellular therapeutic function of antibody against viruses. As it has been known that once infected by DENVs, the released single-stranded RNAs (ssRNAs) will be replicated by the host cell machinery. Viral proteins are then synthesized by the host cells using the viral ssRNAs as templates. One possible anti-viral mechanism of the intracellular HuMAbs is that these affinitive proteins bind to the being translated viral proteins, the envelope protein domain III or domain II for this case^30^, thus halting an assembling of protein components into complete viruses (Fig. 5d mechanism 1). Another possible mechanism is that after viruses have fully been assembled inside the cells, intracellular antibodies bind to them in the similar fashion to the binding when they are outside of the cells (Fig. 5d mechanism 2). Viral inhibition is more effective when HuMAbs are confined inside the infected cells because the viral production site is being blocked. When HuMAbs are limited to only the outside of the infected cells, more viruses can always be produced and secreted out from the infected cells. As a result, ability to bring functional antibody into cells to perform viral neutralization intracellularly, gave a better viral neutralization efficacy.

## Methods

### Synthesis of oxidized carbon black particles (OCBs)

OCBs were prepared by directly oxidizing commercially available carbon black^15^. Briefly, CB (0.1, 0.3 or 0.5 g, Denka Company, Denki Kagaku Kogyo Kabushiki Kaisha, Japan) was mixed with 1.0 g of NaNO_3_ and 50 mL of 18 M H_2_SO_4_, and the mixture was sonicated at 40 kHz at room temperature for 1 h. Next, KMnO_4_ (6.0 g) was slowly added into the mixture with stirring for 90 min (extreme caution must be paid at this step to avoid over heating). After that, 100 mL of water was added and stirred for 30 min. Then, 300 mL of water was added and the mixture was stirred for 10 min. The reaction was stopped by adding 5% (w/v) H_2_O_2_ (50 mL) under stirring at room temperature for 30 min. Finally, the obtained mixture was washed with water under high speed centrifugation (20,000 rpm, for 20 min, 3 times), and the pellet was collected. The pellet was re-suspended in water and dialyzed against water until pH 5.5 (using CelluSep T4, MWCO of 12,000−14,000 Da, Membrane Filtration Products, USA).

### Penetration of OCBs into cell-sized liposome

DOPC (Avanti Polar Lipid, Alabama, USA) solution in chloroform (2 mM, 200 µL) was mixed with glucose solution in methanol (10 mM, 120 µL) in a glass test tube. Then, the mixed solution was dried under nitrogen gas flow to make a thin film. The dried film was kept under vacuum for at least 3 h. After that, 2 mL of water was added and the solution was kept at 37 °C for 3 h in order to hydrate the film and allow the formation of cell-sized liposomes. To start the experiment, the obtained liposome suspension in water was mixed with flu-OCBs. The final concentration of liposomes was controlled at 0.25 mM of lipids. The final concentration of flu-OCBs was at 100 µg/mL OCBs with 30 µg/mL of flu moiety. In the control experiment, liposome suspension in water was mixed with flu solution (in 5% of DMF and 95% water) at the final concentration of 30 mg/mL. After mixing, the suspension was dropped onto the glass slide with a silicon chamber. Then the liposomes in the suspension were observed by CLFM at the λ_ex_/λ_em_ of 488/520 nm^16,17^.

To prepare cell-sized liposomes filled with anthocyanin, thin film of DOPC was prepared by the same method as the unfilled liposome preparation (above), however, instead of adding water to hydrate the lipoid film, 2 mL of 1000 μg/mL anthocyanin solution in water was added and the solution was kept at 37 °C for 2–3 h to hydrate the film and allow the formation of cell-sized liposomes containing anthocyanin inside the vesicle. In order to eliminate anthocyanin at the outside of the liposomes, the liposome suspension was left undisturbed at 37 °C for overnight to allow sedimentation. Then, the aqueous solution above the settled down liposomes was carefully removed, followed by an addition of the same volume of water^16^. Experiment was started by adding OCBs suspension to the anthocyanin-filled liposomes to give the final concentration of lipids and OCBs of 0.25 mM and 100 µg/mL, respectively. The liposome suspension was immediately observed using CLFM at the λ_ex_/λ_em_ of 488/525 nm. Similar observation was carried out on the control anthocyanin-filled liposomes with no OCB (water was added in place of OCBs suspension). Observation was carried out as a function of incubation time.

### Cellular delivery of protein by OCBs

CaSki cell was maintained in Roswell Park Memorial Institute medium 1640 (RPMI 1640 medium) with 2.05 mM L-glutamine (Hyclone Laboratory, Inc., Logan, UT, USA). Cells were incubated at 37 °C for 24 h in humidified atmosphere (5% CO_2_). CaSki cells, at the density of 2 × 10^6^ cells per well, were seeded in 6-well plates on cover slips and incubated at 37 °C for 24 h in humidified atmosphere (5% CO_2_). Each sample which included PBS (negative control), flu-lysozyme, flu-lysozyme mixed with, was added to cells, to give a lysozyme protein final concentration of 10 ppm and OCBs final concentration of 10 ppm. The plate was left for 4 h at 37 °C in a humidified atmosphere (5% CO_2_). After that, cells were fixed by 4% paraformaldehyde and nucleus-stained with DAPI before being subjected to CLFM analysis. Control experiment in which neither flu-lysozyme nor cou-OCBs was added, was also carried out.

Intra cellular trafficking of lysozyme protein in the presence of OCBs was monitored in CaSki cells. CaSki cells were seeded in 8-well chamber (Lab-Tek II Chambered Cover glass, NUNC, NY, USA) at the density of 2 × 10^6^ cells per well, then 50 µl of early endosome fluorescent dye reagent (cellLight™ early endosome-RFP, Bacmam 2.0, Invitrogen, USA) was added and the mixture was incubated overnight at 37 °C in humidified atmosphere (5% CO_2_), after that, 50 µL of lysotracker deep red reagent (in anhydrous DMSO, Lysotracker and Lysosensor probe, Invitrogen, USA) was added (final concentration of lysotracker was 200 nM) and the mixture was incubated for another 2 h at 37 °C. Then, 25 µL sample (flu-lysozyme mixed with cou-OCB), at the same concentrations of lysozyme protein and OCBs of 100 ppm) was added directly to each well (to give the final concentration of the lysozyme protein and OCBs in the cell suspension of 10 ppm). After that the live-cells were immediately monitored for 4 h (pictures recorded every 15 min) under CLSM (FV10i-LIV with universal Plan Super Apochomat 60× phase contrast water immersion objective Lens, Olympus, Tokyo, Japan). Excitation was carried out at 405, 473, 559 and 635 nm (MellesGriot Laser, Carlsbad, CA, USA) and emission was monitored at 450, 520, 584 and 668 nm for cou-OCBs, flu-lysozyme, early endosome specific RFP dye and lysosome specific deep red dye, respectively, using two PMTs that automatically optimized for the detection bandwidth of the four fluorophores. The control cells were prepared in two conditions by the same protocol as mention above. The first control condition, the cells were incubated with endosome fluorescent dye and lysotracker deep red reagents (without cou-OCB and flu-lysozyme). In the second control condition, the cells were incubated with only flu-lysozyme (final concentration of flu-lysozyme in the cell suspension of 10 ppm, no endosome fluorescent dye and lysotracker deep red reagents). Data were processed with FLUOVIEW 3.0 software.

### Antibody delivery

The HuMAbs were produced from Human hybridoma cells which have been prepared through the fusion of human Peripheral Blood Mononuclear cell (PBMC) with SPYMEG myeloma cells, as previously described^31^. flu-HuMAbs was prepared as follows. Fluorescein-5-isothiocyanate (1 mg) was dissolved in 0.1 M Na_2_HPO_4_ solution (2 mL). HuMAbs (50 µL of 2 mg/mL) was mixed with 12.5 µL of 0.2 M Na_2_HPO_4_ solution and 60 µL of the prepared fluorescein solution. Then pH of the mixture was measured and the pH was adjusted to pH 9.5 by adding 0.1 M Na_3_PO_4_ solution. Finally, flu-HuMAbs were dialyzed against phosphate buffered saline (PBS).

Vero cells were maintained in minimum essential medium with Earle’s balanced salts with L-glutamine (MEM/EBSS) and 10% fetal bovine serum (FBS). Vero cells were seeded at the density of 7.5 × 10^4^ cells per well, in the 8 well-chamber slide and allowed to attach for 16–24 h. Next, cells were washed with PBS and then added flu-HuMAbs (final concentration of 64 µg/mL) with or without OCBs (final concentration of 15 μg/mL) in MEM/EBSS with 1% FBS (250 µL). After incubation at 37 °C for appropriate time, cells were washed twice with PBS and then fixed in a 4% paraformaldehyde in PBS. Cells were stained with DAPI. Finally, cells were observed under CLFM.

### Virus neutralization assessment

Vero cells were seeded at the density of 2.5 × 10^4^ cells per well in 96-well-microplate and allowed to attach for 16–24 h. Then, cells were washed with PBS and then infected with 100 FFU of individual DENV serotypes in MEM/EBSS (50 µL). After incubation at 37 °C for 1 h, culture media was removed and HuMAbs with or without OCB (final concentration of 15 ppm) in MEM/EBSS with 1% FBS (50 µL) were added. After incubation at 37 °C for 2 h, 100 µL of a mixture of 2X minimum essential medium: 2% carboxymethyl cellulose (1:1) with 2.5% FBS were added. And then, cells were incubated at 37 °C for 2 or 3 days.

Indirect immunofluorescence (IF) assay was successively conducted on the cells from neutralization assay. Cells were fixed with 3.7% formaldehyde in PBS and permeabilized with 0.1% Triton X-100 in PBS. After that, cells were incubated with hybridoma culture fluids (primary antibody, for HuMAbs blocked DENVs). Finally the bound antibody was visualized by further reaction with an Alexa Fluor 488 goat anti-human IgG (H + L), for a cross-adsorbed secondary antibody (1:1,000). These assays were performed in triplicated (see ‘Supplementary Information’ for more information)^19^.

### Data Availability

All data generated or analyzed during this study are included in this published article and its Supplementary Information files.

## Electronic supplementary material

Supplementary Information
