# Supplementary material for: Oxidized Carbon Black: Preparation, Characterization and Application in Antibody Delivery across Cell Membrane
Source: Sci Rep. 2018 Feb 6;8:2489. doi: 10.1038/s41598-018-20650-4 (PMC5802750; doi:10.1038/s41598-018-20650-4)
Supplement: Supplementary file 1 — Supplementary Information [file 41598_2018_20650_MOESM1_ESM.doc]

Supplementary Information

Oxidized Carbon Black: Preparation, Characterization and Application in Antibody Delivery across Cell Membrane

Kittima Amornwachirabodee,^1,2,+^ Nattapol Tantimekin,^1,+^ Porntip Pan-In,^1,3^ Tanapat Palaga,^4^ Prompong Pienpinijtham,^1^ Chonlatip Pipattanaboon,^5^ Thanyada Sukmanee,^1^ Patcharee Ritprajak,^6^ Promchat Charoenpat,^6^ Pannamthip Pitaksajjakul,^7^ Pongrama Ramasoota ^7^ and Supason Wanichwecharungruang^1,7,*^

^1^ Department of Chemistry, Faculty of Science, Chulalongkorn University, Bangkok, 10330, Thailand

^2^ Center of Excellence on Petrochemical and Materials Technology, Chulalongkorn University, Bangkok, 10330, Thailand

^3^ Nanotec-Chulalongkorn University Center of Excellence on Food and Agriculture, Chulalongkorn University, Bangkok, Thailand

^4^ Department of Microbiology, Faculty of Science, Chulalongkorn University, Bangkok, 10330, Thailand

^5^ Center of Excellence for Antibody Research, Faculty of Tropical Medicine,

Mahidol University, Bangkok, 10400, Thailand

^6^ Department of Microbiology, and RU in Oral Microbiology, Faculty of Dentistry, Chulalongkorn University, Bangkok, 10330, Thailand

^7^ Center of Excellence for Antibody Research, and Department of Social and Environmental Medicine, Faculty of Tropical Medicine, Mahidol University, Bangkok, 10400, Thailand

^8^ Center of Excellence in Materials and Bio-Interfaces, Chulalongkorn University, Bangkok, 10330, Thailand

*supason.p@chula.ac.th

^+^ These authors contribute equally.

**Supplementary Figures**

**
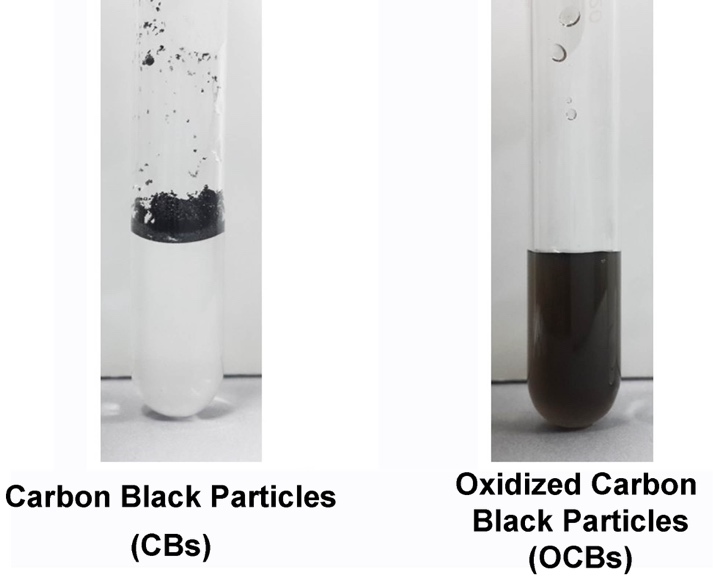
**

**Figure S1.** Water suspensions of carbon black (CBs) and oxidized carbon black (OCBs).


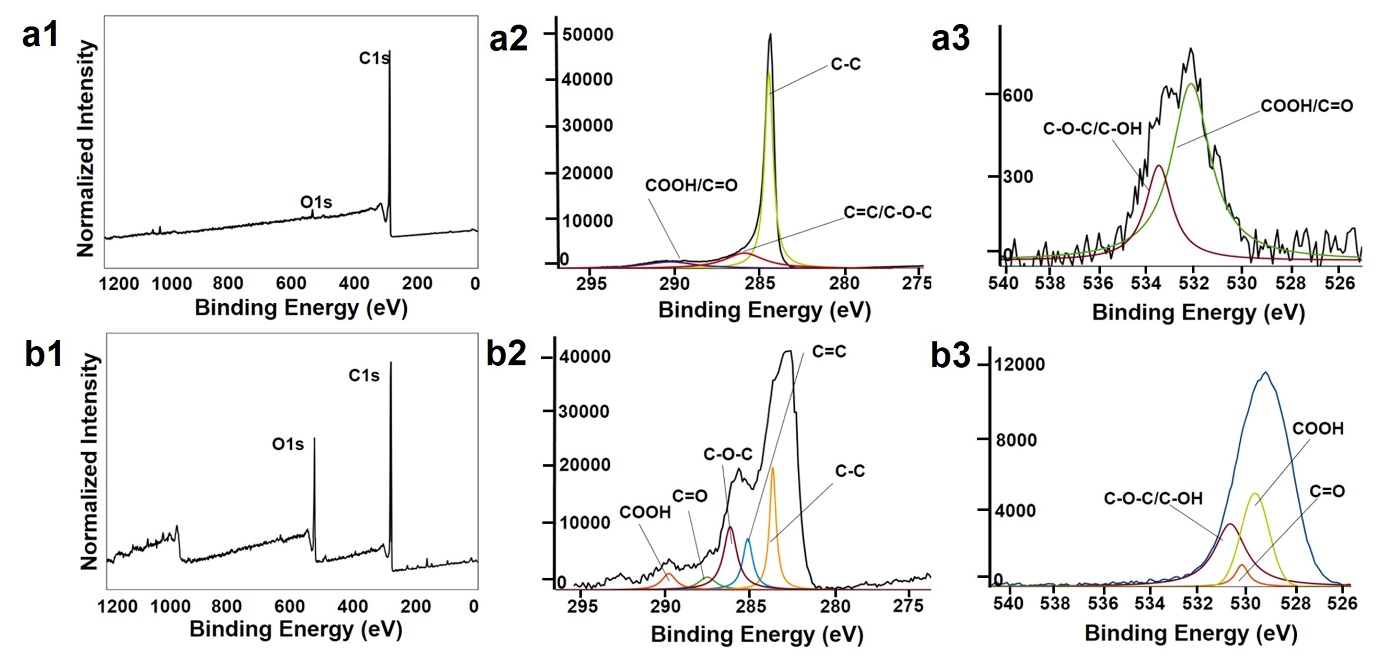


**Figure S2.** XPS spectra of CB and OCBs. Survey Scan spectra (a1 and b1), deconvoluted C1s fitting spectra (a2 and b2) and deconvoluted O1s fitting spectra (a3 and b3) of CBs (a1, a2, a3) and OCBs (b1, b2, b3).


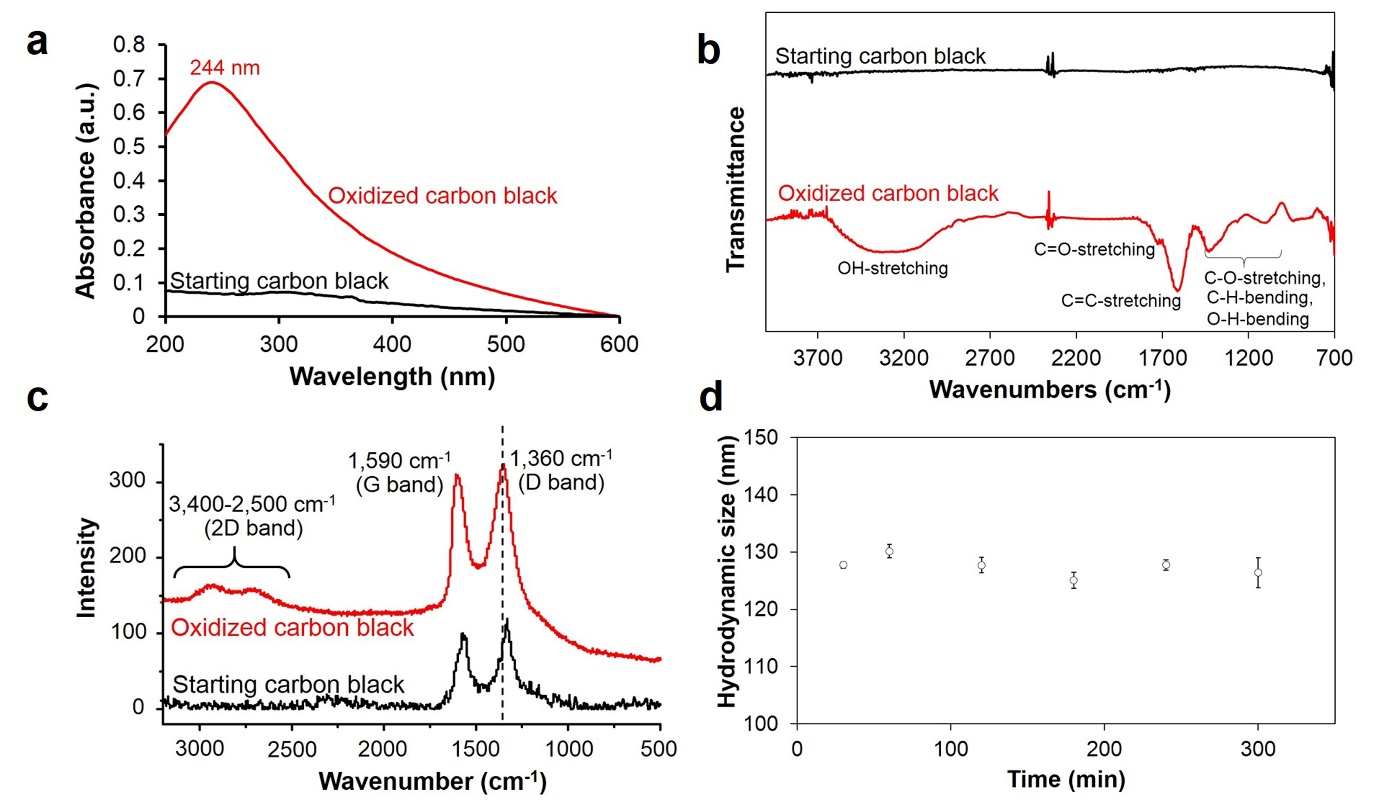


**Figure S3.** OCBs characteristics. (a) UV absorption spectra, (b) FTIR spectra, (c) Raman spectra of CBs and OCBs, and (d) Average hydrodynamic size (shown as mean ± SD) of OCBs at various sitting times.


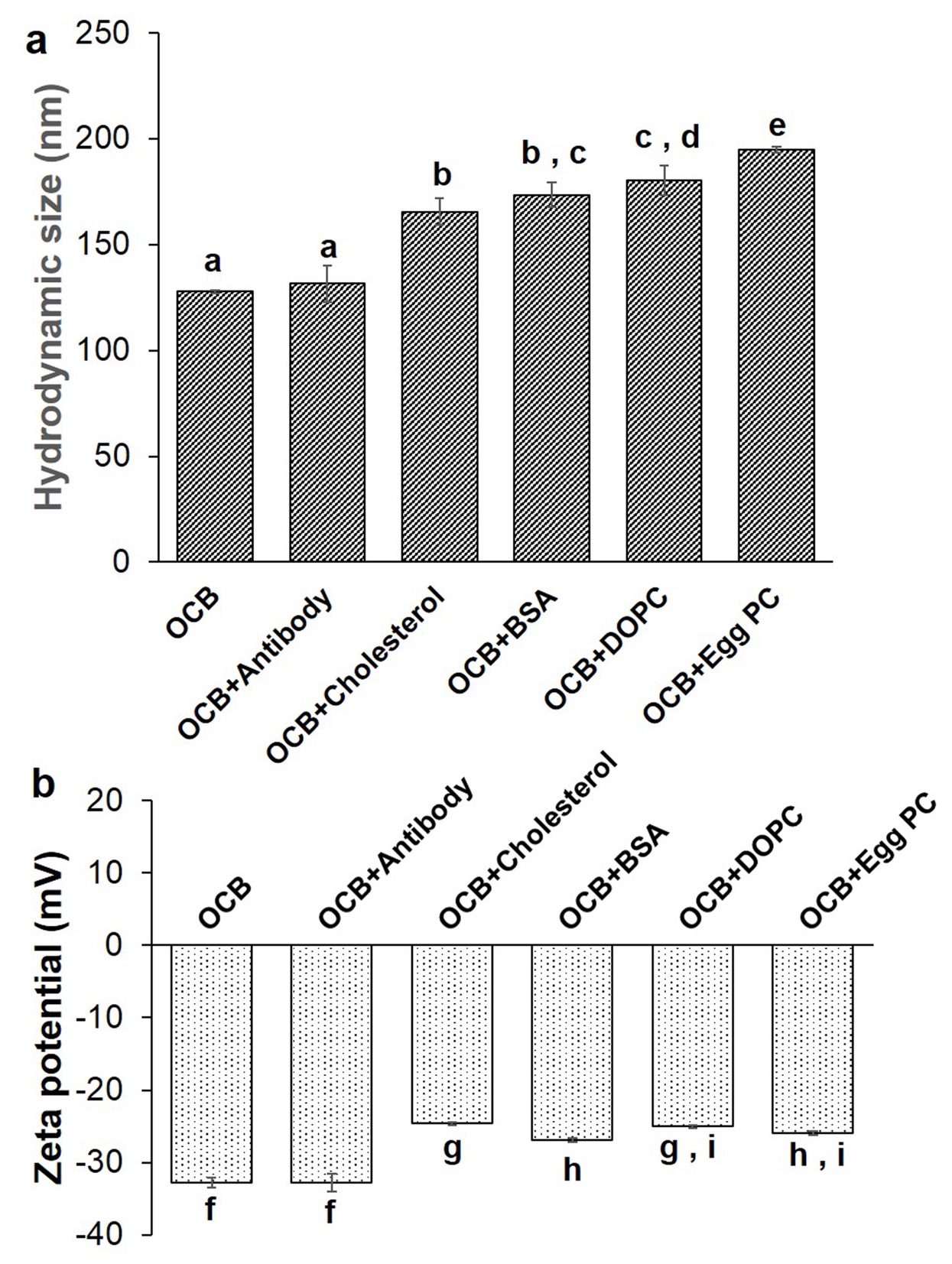


**Figure S4.** Hydrodynamic sizes (a) and zeta potentials (b) of OCBs before and after incubation with various molecules. Data are shown as the mean ± SD and different alphabets on the data bars indicate significant statistical difference (same alphabet means “not significant different”), as determined by one way ANOVA Tukey at P< 0.05.


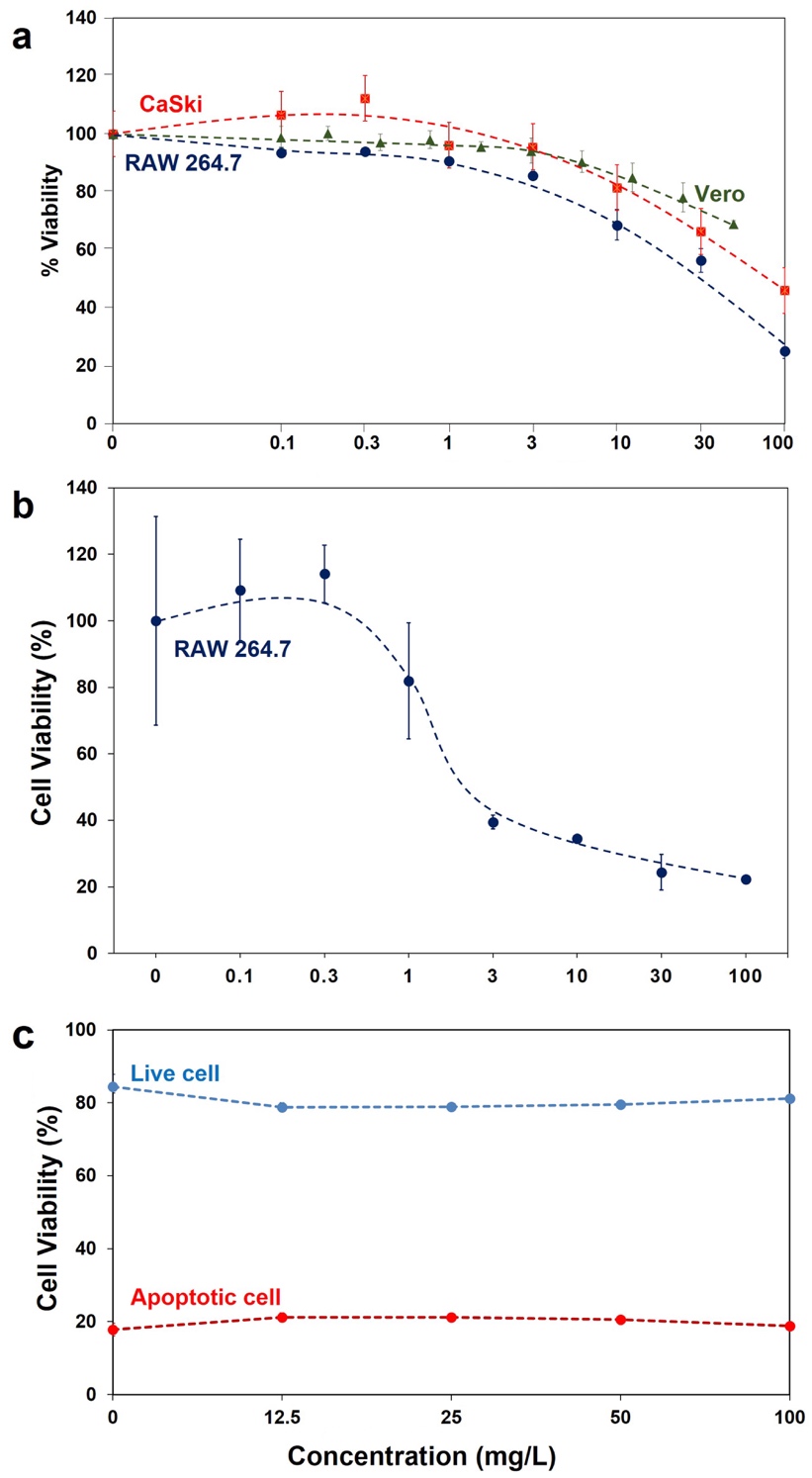


**Figure S5.** In vitro cytotoxicity of OCBs. (a) RAW 264.7, CaSki and Vero cells after a 48 h exposure to various concentrations of OCBs, as evaluated by an MTT assay. (b) RAW 264.7 cells after 7 days of exposure to various concentrations of OCBs, as evaluated by an MTT assay. (c) Bone marrow-derived primary dendritic cells, BM-DCs when exposed to various concentrations of OCBs, as evaluated by flow cytometric analysis of an apoptosis detection; Annexin V^−^ 7AAD^−^ represented live cells, and Annexin V^+^ 7AAD^+^ represented apoptotic cells. Data are shown as the mean ± SD and are derived from three independent repeats.


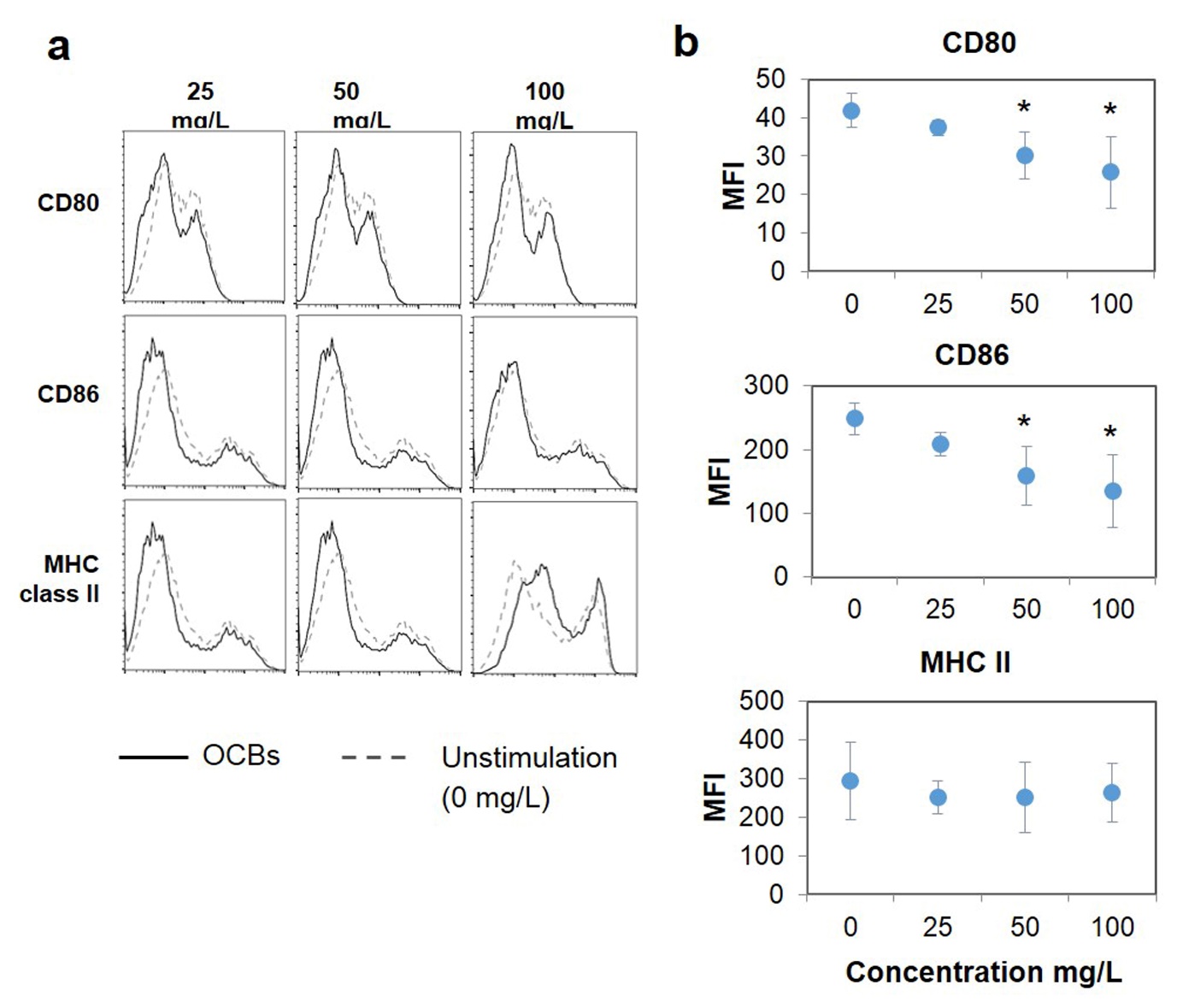


**Figure S6.** In vitro immunogenicity test of OCBs in primary dendritic cells. BM-DCs were stimulated with various concentration of OCBs as indicated and the expression of the activation marker CD80, CD86 and MHC class II were determined by flow cytometric analysis. (a) CD11c^+^ dendritic cells were gated and the fluorescence intensity of the activation markers was shown as the histograms. (b) Mean fluorescent intensity (MFI) of the activation markers on CD11c^+^ dendritic cells. Data are shown as the mean ± SD and are derived from three independent repeats (N=5). *P<0.05 when compared to 0 mg/L OCBs.

**Supplementary Table**

**Table S1.** Morphology and size of the oxidized carbon black nanoparticles (OCBs) prepared under different oxidation conditions.

| **Sample** | **CB:KMnO_4_ Weight ratios** | **SEM**  **size*^a^***  **(nm)** | **TEM**  **size*^a^***  **(nm)** | **DLS** | | |
| --- | --- | --- | --- | --- | --- | --- |
|  |  |  |  | **Hydro-dynamic size (nm)** | **PDI** | **Zeta potential (mV)** |
| OCBs-1 | 0.3:6.0 | 129 ± 25.6 | 115 ± 21.4 | 127 ± 0.51 | 0.18 | -33 ± 1.0 |
| OCBs-2 | 0.5:6.0 | 200 ± 51.5 | 228 ± 86.5 | 255 ± 2.17 | 0.33 | -34 ± 1.5 |

*^a^*Average particle sizes were obtained by ImageJ analysis. At least 50 particles in each image and three images from 3 independent experiments were used for each material.

**Supplementary Methods**

**Characterization of OCBs**

The obtained OCBs were characterized by *(i) scanning electron microscope* (SEM, JEOL JSM-6400, Tokyo, Japan, using an accelerating voltage of 15 kV), *(ii) transmission electron microscope* (TEM, JEOL JEM-2100, Tokyo, Japan, using an accelerating voltage of 120 kV in conjunction with selected area electron diffraction), *(iii) dynamic light scattering* (DLS, Malvern Zetasizer nanoseries model S4700, using a He−Ne laser beam at 632.8 nm and scattering angle of 173°), *(iv) ATR-FTIR* (Nicolet 6700, Thermo Scientific, Thermo Fisher Scientific Inc., MA, USA ), *(v) DXR Raman microscope* (Thermo Scientific, Thermo Fisher Scientific Inc., MA, USA, using a 10X objective and a 5 mW diode laser (λ= 780 nm) excitation source, *(vi) X-ray photoelectronic spectroscopy* (XPS, Kratos AXIS Ultra DLD instrument, Kratos, Manchester, England, using a monochromatic Al KαX-ray source at 1486.6 eV and operated at 150 W, 15 kV and 10 mA, high resolution spectra (C1s and O1s) acquired using a pass energy of 20 eV with 0.1 eV energy step, using C1s peak at 285 eV as referenced binding energy), *(vii) elemental analysis* (EA, combustion through the PE2400 Series II, Perkin-Elmer, MA, USA), and *(viii) UV-visible absorption analysis* (Shimadzu Corp., Kyoto, Japan).

**Fluorescence Dye Labelling on OCBs and Protein**

Fluorescence dye moieties with carboxyl group (5-carboxyfluorescein or fluorescein, coumarin-3-carboxylic acid or coumarin, and 5-carboxytetramethylrhodamine or TAMRA, purchased from Life Technologies, Carlsbad, CA, USA) were grafted onto the oxidized carbon black particulates through the coupling reaction using 1-ethyl-3-(3-dimethylaminopropyl)-carbodiimide (EDCI, Acros Organics, Belgium) and N-hydroxysuccinamide (NHS, Acros Organics, Belgium). Firstly, 2.2 mg of EDCI in water (1.0 mL) was slowly added into aqueous suspension of the oxidized carbon black particulates (400 ppm, 5 mL) at 0 °C, under N_2_ atmosphere. The mixture was stirred for 30 min. After that, 1.3 mg of NHS in water (1.0 mL) was dropped into the reaction, followed by an addition of fluorescence dye solution in DMF (2000 ppm, 0.5 mL). The reaction was stirred overnight. All products were purified by dialysis (pre-swollen membrane, CelluSep T4, MWCO of 12,000−14,000 Da). Hen egg white lysozyme protein (MW of 300 kDa, purchased from Sigma Aldrich, St Louis, MO, USA) was labelled with fluorescein. Firstly, 4.4 mg of 5-carboxyfluorescein in DMF (0.5 mL) was reacted with 5.7 mg of EDCI in water (1.0 mL). The mixed suspension was further stirred at 4 ^o^C for 30 min. After that, 5.7 mg of NHS in water (1.0 mL) was dropped into the reaction, followed by an addition of 25 mg of lysozyme protein in water (1.0 mL). The reaction was stirred at 4 ^o^C overnight. Then the product was purified at 4 ^o^C by dialysis (pre-swollen membrane, CelluSep T4, MWCO of 12,000−14,000 Da).

**Adsorption of Phospholipids on OCBs**

Changes in sizes and zeta potential of the OCBs after being incubated with different tested materials were investigated. OCBs were incubated with each of the tested materials which include cholesterol, DOPC, L-α-phosphatidylcholine (Egg PC), Bovine serum albumin (BSA) and antibody (HuMAbs), at the ratio of OCB to tested material of 1:2 (w/w). The mixture was incubated for 2 h, after that the non-adsorbed molecules were removed by centrifugation (20,000rpm, 15 min) at 4 ^o^C. The precipitate was re-dispersed in water, then the size and zeta potential were measured by DLS. The results were analyzed by SPSS program using one-way ANOVA method.

**In vitro Cytotoxicity of OCBs**

RAW 264.7 cell was maintained in Dulbecco’s modified Eagle medium (DMEM) (HyClone, Logan, UT, USA) supplemented with 10% (v/v) of fetal bovine serum (FBS), 1% (w/v) sodium pyruvate (HyClone), 1% (w/v) N-2-hydroxyethylpiperazine-N’-2-ethanesulfonic acid (HyClone) (HEPES), 100 U/mL penicillin and 0.4 mg/mL streptomycin sulphate. CaSki cell was maintained in Roswell Park Memorial Institute medium 1640 (RPMI 1640 medium) with 2.05 mM L-glutamine (Hyclone Laboratory, Inc., Logan, UT, USA). Cells were incubated at 37 °C for 24 h in humidified atmosphere (5% CO_2_). Vero cell (ATCC) was maintained in minimum essential medium with Earle’s balanced salts with L-glutamine (MEM/EBSS) and 10% fetal bovine serum (FBS). Cytotoxicity OCBs was acquired by MTT assay using RAW 264.7 and CaSki and Vero cells. The cells were seeded into a 96 well plates at density of 1×10^4^ cells in culture medium. After removal of the culture medium, cells were incubated with OCBs at concentrations of 0.1-100 mg/L in culture media, for 48 h. After incubation, 10 μL of PBS containing 1 mg/mL MTT solution was added to each well and the plates were incubated for 4 h at 37 °C. After that culture media was removed from the wells and isopropanol (200 μL/well) was added to dissolve formazan crystals. Plates were spin at 4839.77 g for 10 min, then 50 μL of the supernatant was collected and subjected to absorbance measurement at 540 nm by microplate reader (Biochrom Anthos 2010, Biochrom Ltd., Cambridge, UK). The cytotoxicity of OCB at 1 week was test with RAW 264.7 cells. The cells were seeded into a 96 well plates at density of 1×10^3^ cells in culture medium and followed by incubated with OCBs at concentrations of 0.1-100 mg/L in culture media for 1 week. Then MTT method was carried out as mentioned above. All conditions were tested in triplicate. Cell viability was calculated using the equation below (Equation 1).


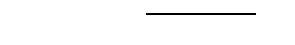
 (Eq.1)

Female Balb/c, 5 weeks old, were purchased from National Laboratory Animal Center, Mahidol University. All procedures were reviewed and approved by the Institutional Animal Care and Use Committee of Chulalongkorn University (Animal protocol 1573019). Bone marrow derived dendritic cells (BM-DCs) were derived from mouse femur bone marrow. Briefly, bone marrow cells were cultured in RPMI (GIBCO,Carlsbad, CA, USA) supplemented with 10% (V/V) FBS (GIBCO), 0.2 mM Glutamax (GIBCO), 100 U/mL penicillin and 0.1 mg/mL streptomycin (Hyclone), 10 ng/mL recombinant murine GM-CSF and IL-4 (Peprotech, Rocky Hill, NJ, USA) at 37 °C in humidified atmosphere (5% CO_2_). At day 7, BM-DCs were incubated with OCBs at the concentration of 12.5, 25, 50 and 100 mg/mL for 24 h and the cytotoxicity of OCBs was evaluated by Annexin V apoptosis detection kit with 7AAD (Biolegend, San Diego, CA, USA). The stained cells were analyzed by flow cytometry (BD FACSCalibur, BD Bioscience, San Jose, CA, USA) and FlowJo software (Tree Star Inc, Ashland, OR, USA).

**In vitro Immuno-Response of OCBs**

The 7 day cultured BM-DC was incubated with OCBs at the concentrations of 25, 50 and 100 mg/mL, for 24 h. The cells were harvested and stained with fluorescence-tagged antibody against mouse CD11c, CD80, CD86 and I-A/I-E (MHC class II) (Biolegend) and analyzed by flow cytometry and FlowJo software.

**Cellular Uptake of OCBs**

RAW 264.7 cells, at the density of 2×10^6^ cells per well, were seeded in 6-well plates on cover slips and incubated at 37 °C for 24 h in humidified atmosphere (5% CO_2_) with DMEM. Each test sample, which included PBS (negative control) and TAMRA-labeled OCBs, was added to cells at the final concentration of 10 ppm. The plate was left for 4 h at 37 °C in a humidified atmosphere (5% CO_2_). Cells were fixed by 4% paraformaldehyde, then they were incubated with 50 µL of 0.01 mg/mL DAPI solution for 10 min (to stain nuclei of cells) before being subjected to CLFM analysis (Nikon Digital Eclipse C1-Si, equipped with Plan Apochromat VC 100×, BDLaser (MellesGriot, Carlsbad, CA, USA), a Nikon TE2000-U microscope, a 32-channel PMT-spectral-detector and Nikon-EZ-C1 Gold Version 3.80 software). Cells were also subjected to CLFM analysis without DAPI incubation. Uptakes of OCBs into human cervical cancer cell (CaSki) was carried out similarly but with different culture media. RPMI and 10 ppm TAMRA-labeled OCBs were used for CaSki. Fluorescence signals of DAPI and TAMRA were detected at λ_ex_/λ_em_ of 405/450 nm and 561/595 nm, respectively.
